# Juvenile idiopathic arthritis: cross-sectional study of incidence and prevalence observed in a tertiary center of spain

**DOI:** 10.1186/1546-0096-12-S1-P173

**Published:** 2014-09-17

**Authors:** Marta Medrano San Ildefonso, Almudena Román Pascual

**Affiliations:** 1 Pediatric Rheumatology, Hospital Universitario Miguel Servet, Zaragoza, Spain; 2 Reumathology, Hospital Alcañiz, Teruel, Spain

## Introduction

Juvenile Idiopathic Arthritis (JIA) is the most chronic musculoskeletal disease of pediatric population. Its incidence and prevalence vary considerably throught the world. The Spanish Society of Rheumatology has estimated a incidence and prevalence of 8-22/100.000 and 0.7-40/100.000 respectively.

## Objectives

Estimate the incidence and prevalence of juvenile idiopathic arthritis (JIA) in a paediatric tertiary care centre of Spain.

## Methods

A 15-years (1997-2012), prospective, population-based study was then carried out to determine the incidence of JIA. Prospective and retrospective data retrieval was performed to calculate prevalence. The International League of Associations for Rheumatology (ILAR, Edmonton revision) classification criteria were applied. Data were compared by Chi-square, student t or wilcoxon test. Significance was set at 5%. Statistical analysis was performed with SPSS version 15.0 software.

## Results

We identified 132 cases of JIA JIA according to ILAR criteria: 81 girls (61.4%) and 51 boys (38.6%). Over the study period, 20 new cases of JIA were diagnosed in Zaragoza. The mean annual incidence was 14.8/100000 children aged less than 16 years. In Aragón 23 new cases cases of JIA were diagnosed so the mean annual incidence was 14.8/100000 and prevalence was 34.64/100.000 children aged less than 16 years.

The mean age was 7.54 years (95 % CI: 6.82-8.25 ) . The most frequent form of onset was persistent oligoarticular arthritis, followed by enthesitis-related arthritis, psoriatic arthritis, rheumatoid factor negative polyarticular arthritis, undifferentiaded arthritis, systemic disease and rheumatoid factor positive polyarticular arthritis.

In boys the most frequent category was been arthritis - enthesitis (n = 28, 54.9 %) and in girls was oligoarticular JIA (n = 55, 67.9 %). The average age at which the arthritis was diagnosed was significantly earlier in the group of patients who of oligoarticular disease (6.4 years) than patients who polyarticular seropositive disease (10.74 years).

## Conclusion

Incidence and prevalence rates are similar than those reported for several countries of Europe. To avoid underestimation of incidence and prevalence, epidemiological studies of JIA should be population-based rather than referral center-based. Further descriptive studies of JCA in different well-defined geographic areas are important to make valid comparisons. Such comparisons could give clues to etiological factors, both genetic and environmental.

## Disclosure of interest

None declared.

